# Supplementary material for: Ultrasonic Enhancement of Aqueous Two-Phase Extraction and Acid Hydrolysis of Flavonoids from Malvaviscus arboreus Cav. Flower for Evaluation of Antioxidant Activity
Source: Antioxidants (Basel). 2022 Oct 16;11(10):2039. doi: 10.3390/antiox11102039 (PMC9598477; doi:10.3390/antiox11102039)
Supplement: Supplementary file 1 [file antioxidants-11-02039-s001.zip › antioxidants-1948647-supplementary.pdf]

Supplementary materials

Supplementary materials included ten figures and one table.

Figures captions:

Figure S1. *Malvaviscus arboreus* Cav. sample of fresh flower (a), Drying flower (b), flower powder by grinding (c).

Figure S2. Extracted ion chromatogram (EIC) of the flavonoid glucosides extracted from MACF by UAATPE.

Figure S3. MS/MS spectra and the fragmentation pattern for catechin glucoside.

Figure S4. MS/MS spectra and the fragmentation pattern for cyanidin glucoside.

Figure S5. MS/MS spectra and the fragmentation pattern for pelargonidin glucosides.

Figure S6. MS/MS spectra and the fragmentation pattern for quercetin glucosides.

Figure S7. MS/MS spectra and the fragmentation pattern for kaempferol glucosides.

Figure S8. Extracted ion chromatogram (EIC) of the main flavonoid aglycones obtained by UAAH.

Figure S9. MS/MS spectra and fragmentation pattern of the main flavonoid aglycones in the hydrolysate.

Figure S10. The chromatograms of the extract before (a) and after (b) hydrolysis by HPLC-DAD detection.

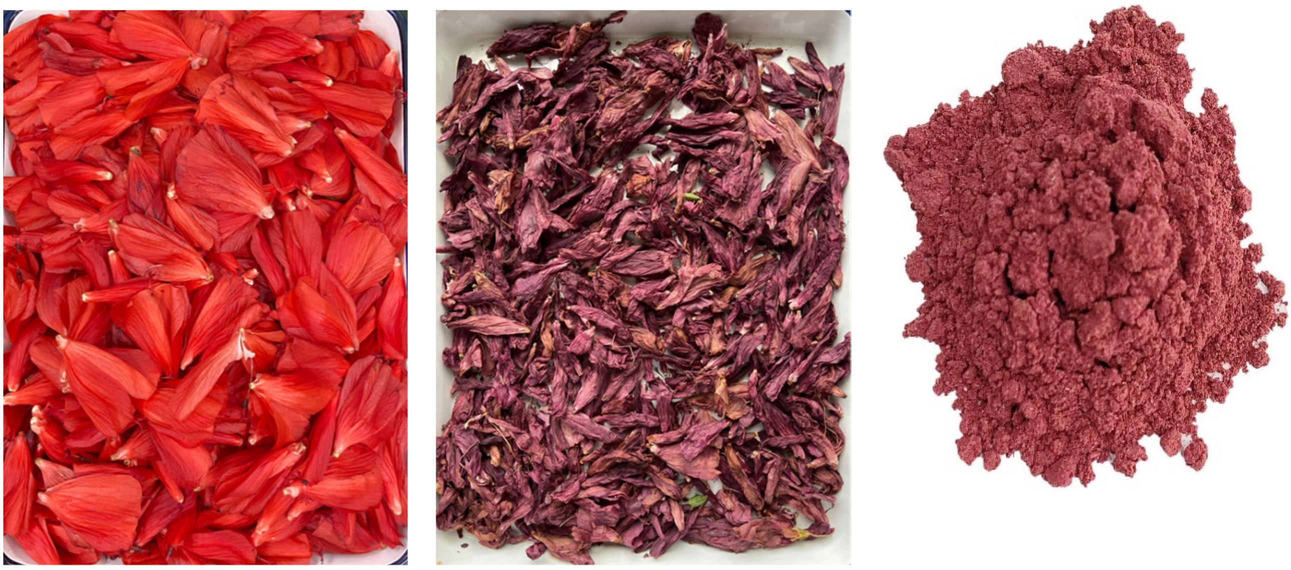

(a) (b) (c)

Figure S1. *Malvaviscus arboreus* Cav. sample of fresh flower (a), Drying flower (b), flower powder by grinding (c).

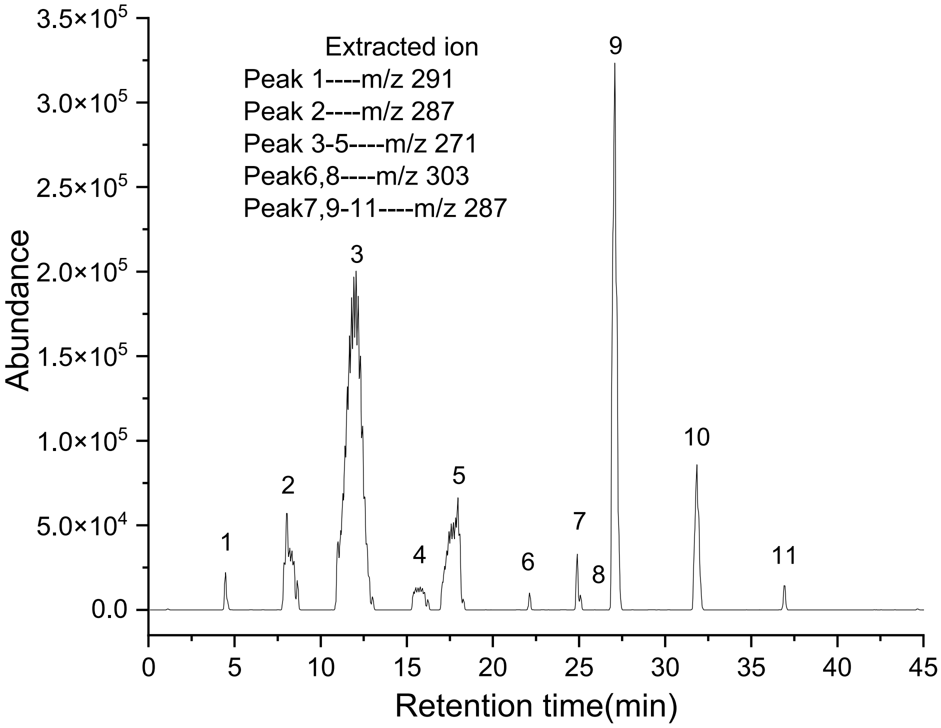

Figure S2. Extracted ion chromatogram (EIC) of the flavonoid glucosides extracted from MAC by UAATPE.

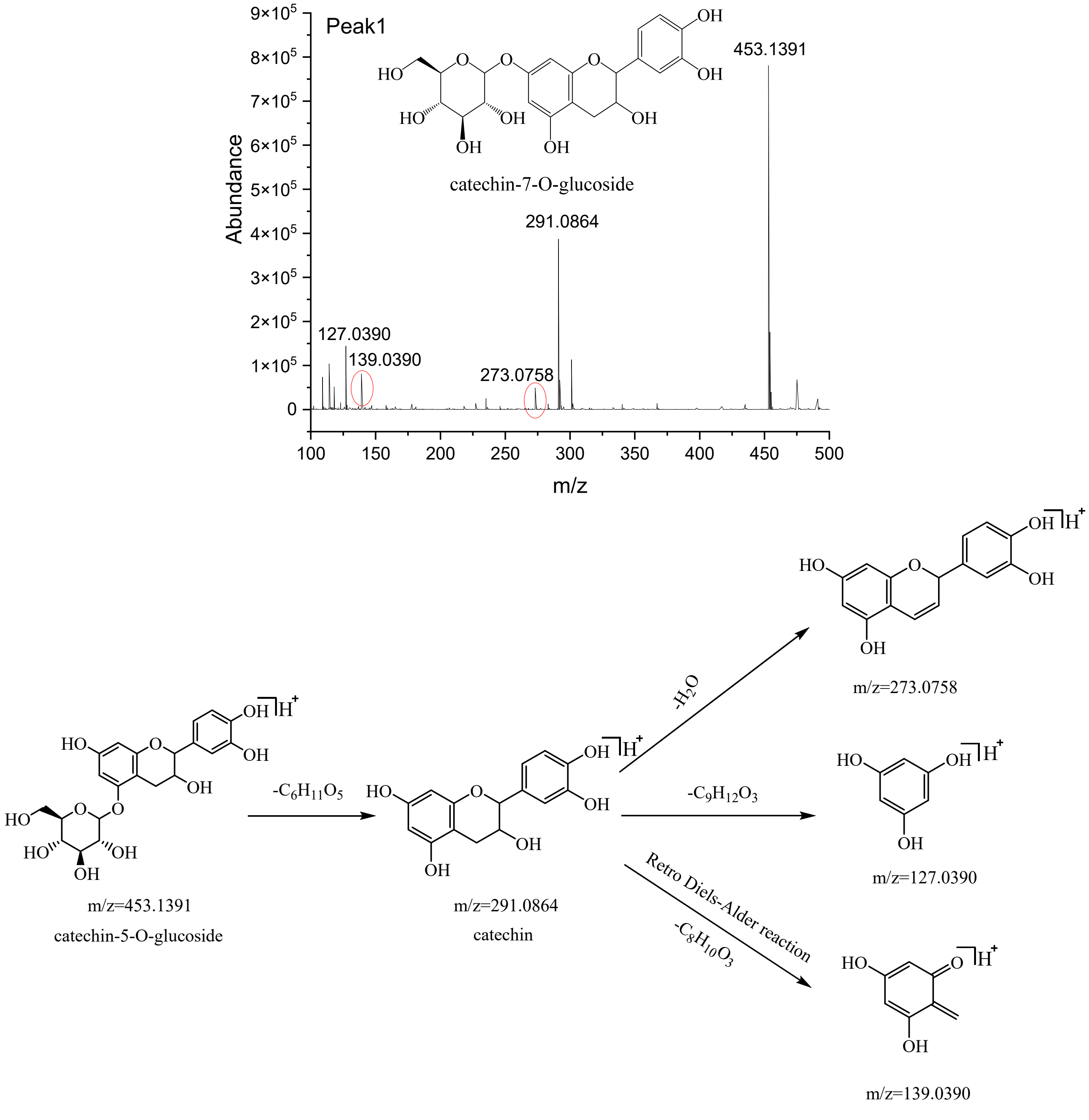

Figure S3. MS/MS spectra and the fragmentation pattern for catechin glucoside.

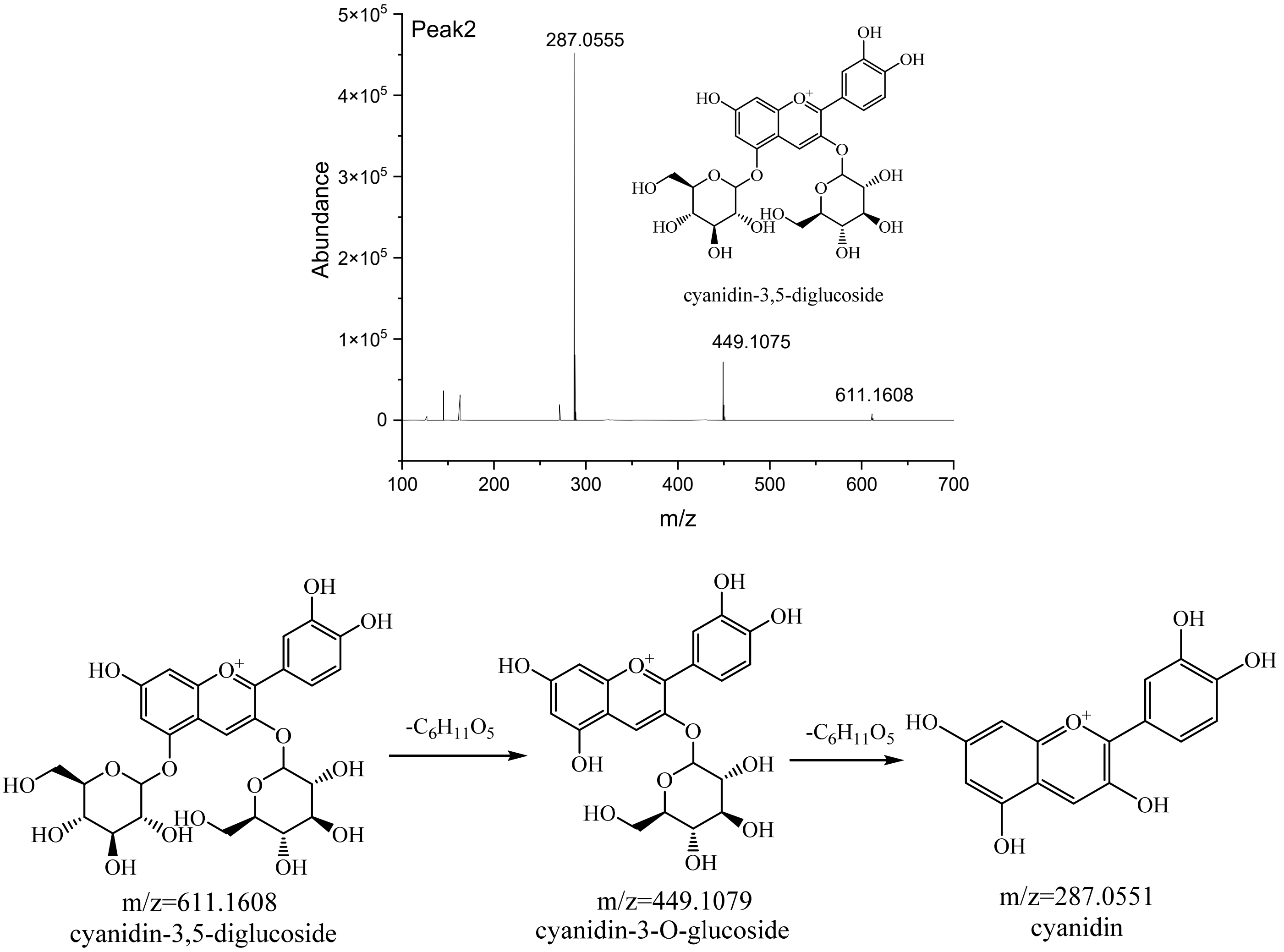

Figure S4. MS/MS spectra and the fragmentation pattern for cyaniding glucoside.

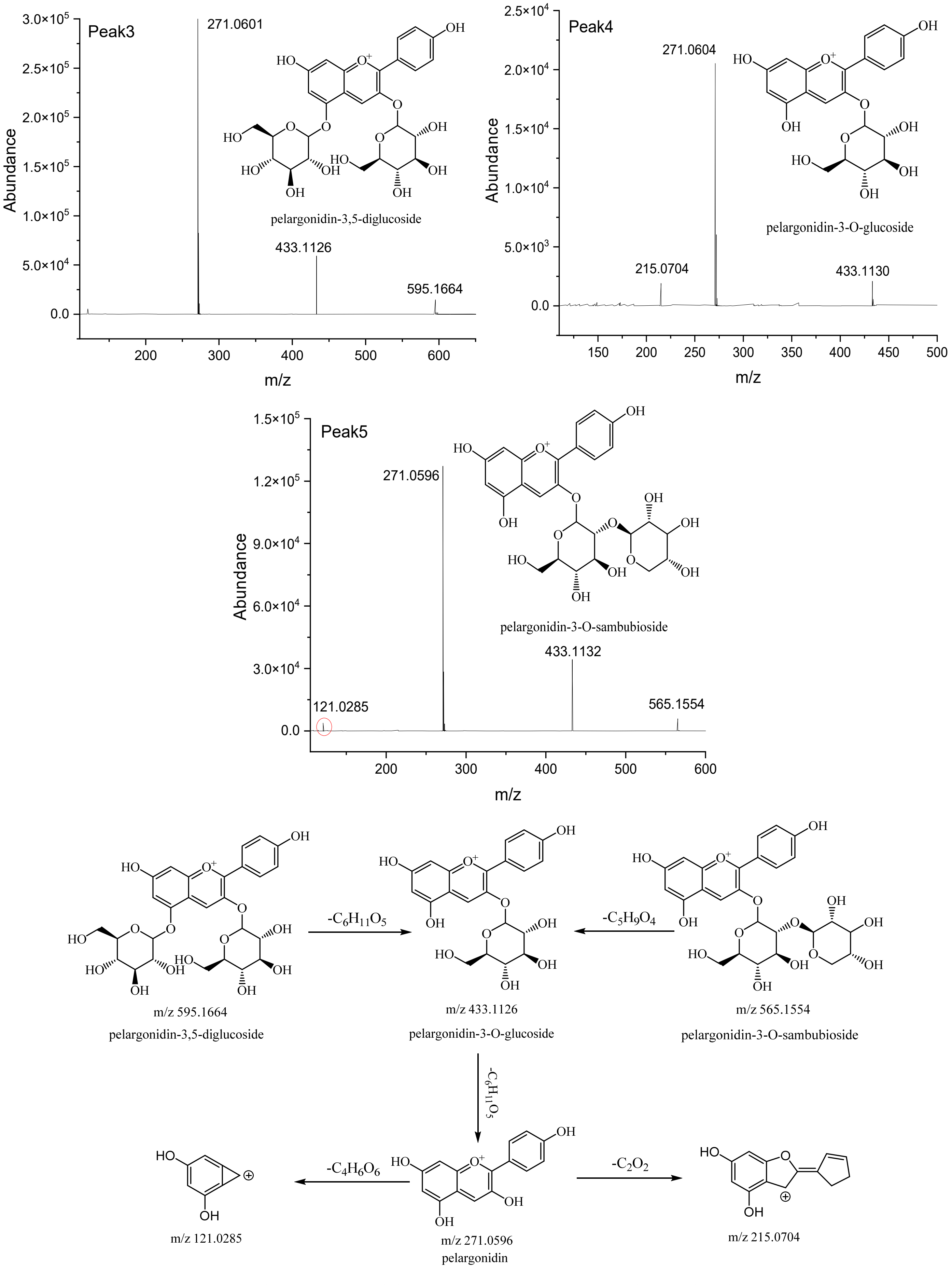

Figure S5. MS/MS spectra and the fragmentation pattern for pelargonidin glucosides.

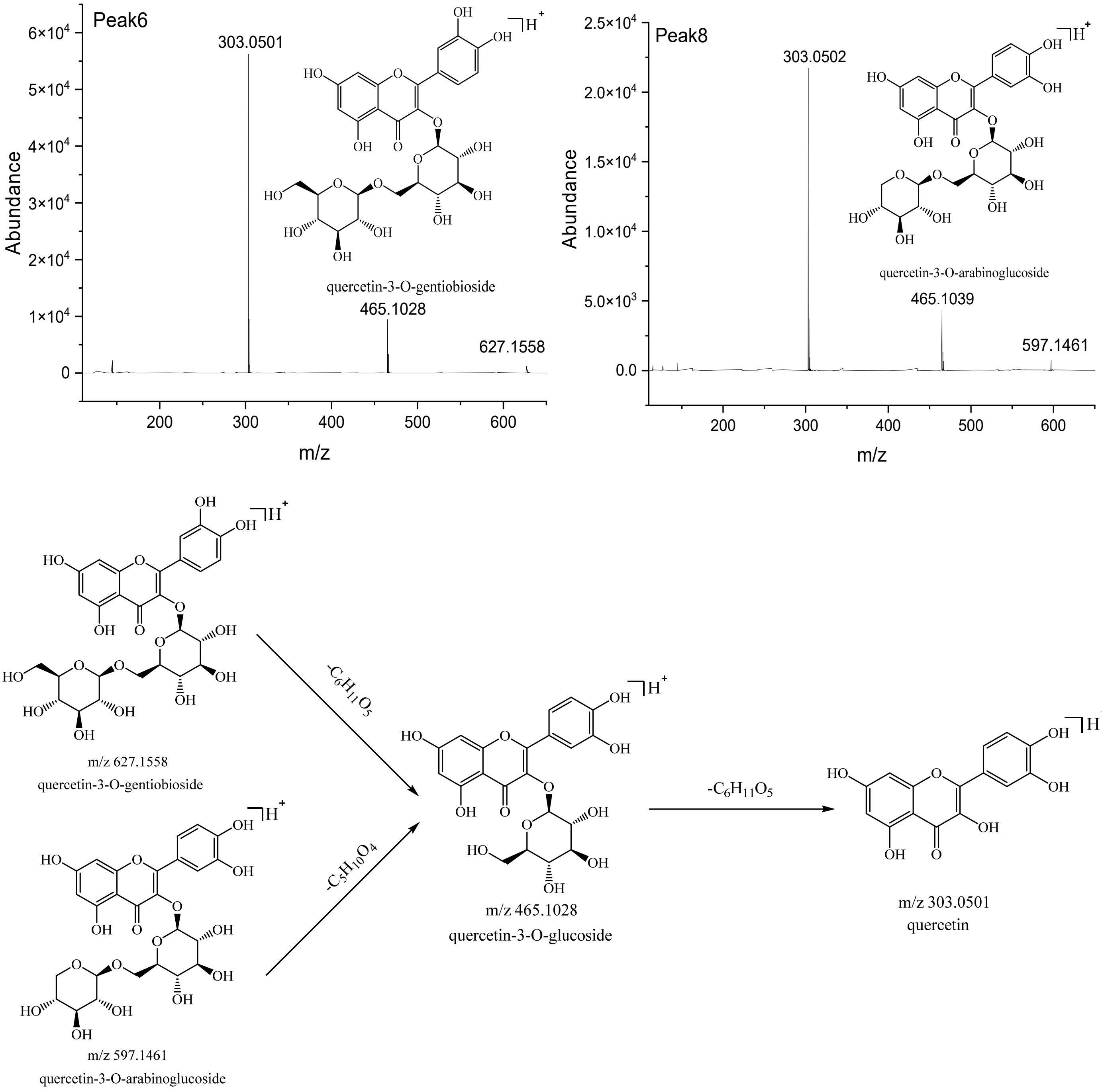

Figure S6. MS/MS spectra and the fragmentation pattern for quercetin glucosides.

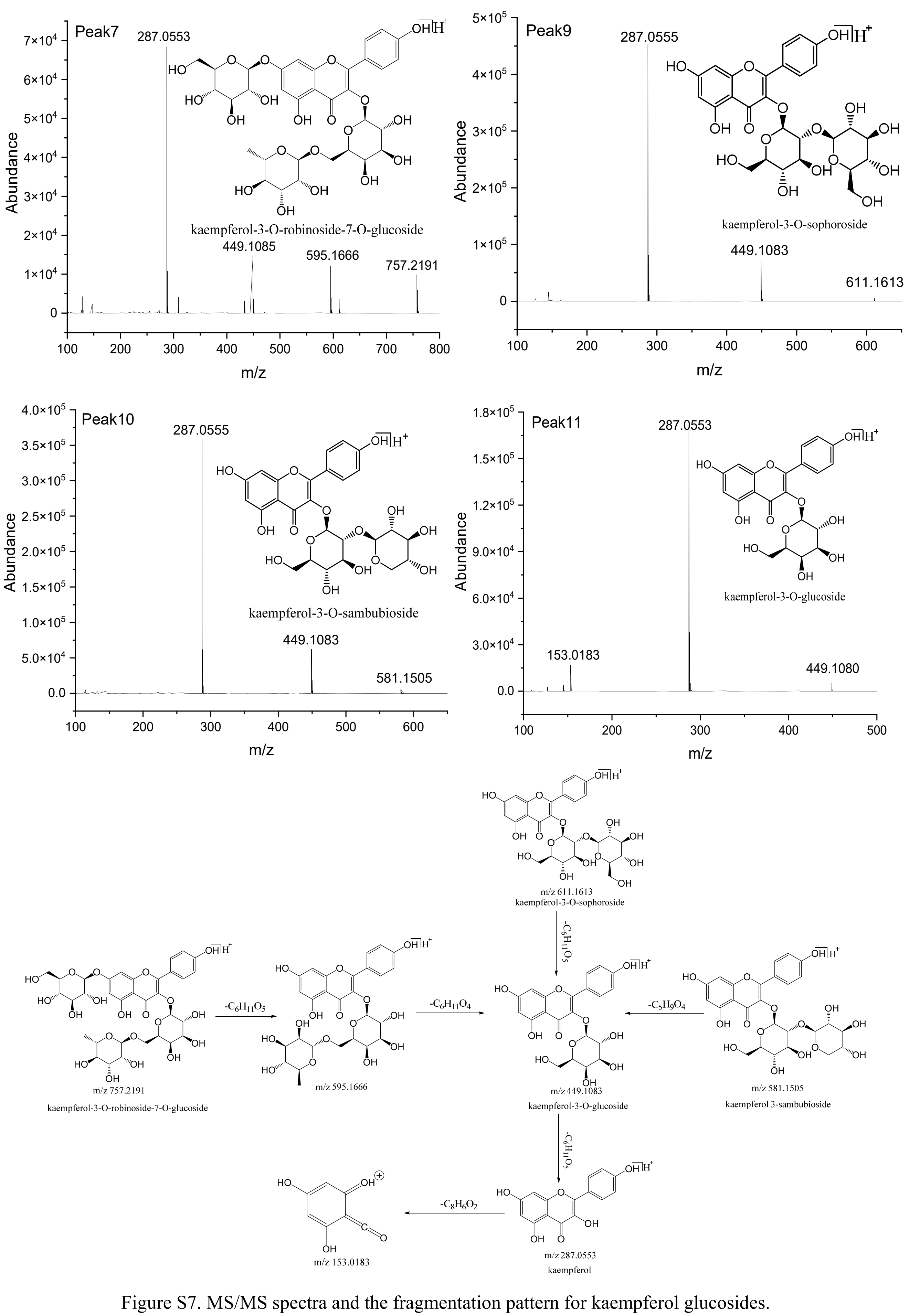

Figure S7. MS/MS spectra and the fragmentation pattern for kaempferol glucosides.

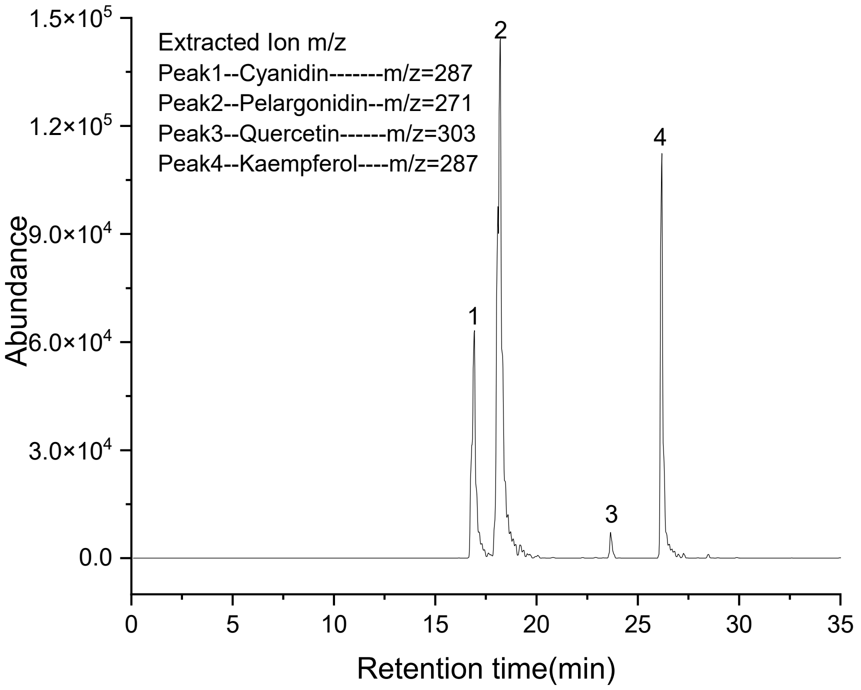

Figure S8. Extracted ion chromatogram (EIC) of the main flavonoid aglycones obtained by UAAH.

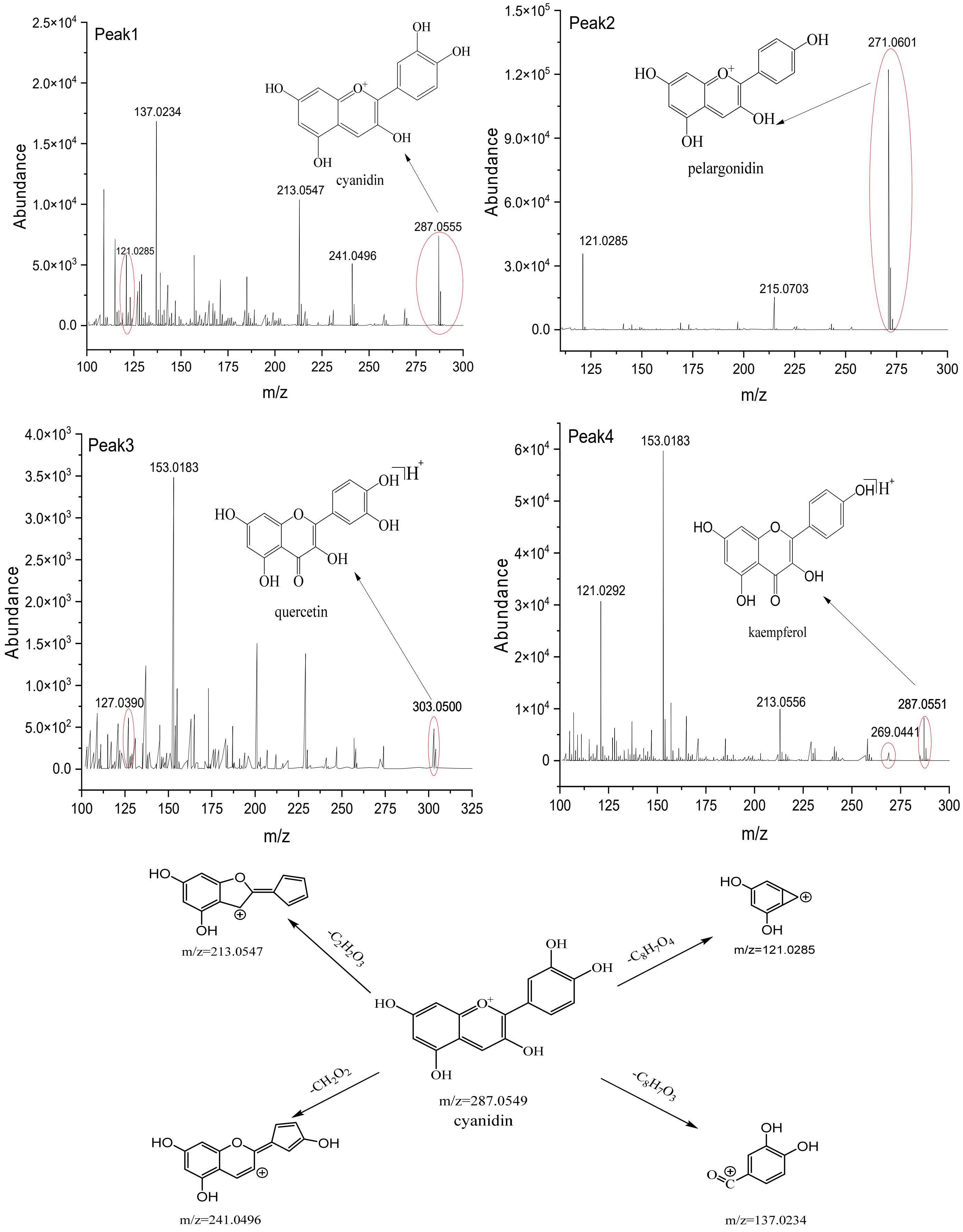

Peak1
Abundance
m/z
137.0234
213.0547
241.0496
287.0555
121.0285
cyanidin
Peak2
271.0601
121.0285
215.0703
pelargonidin
Peak3
153.0183
127.0390
303.0500
quercetin
Peak4
153.0183
121.0292
213.0556
269.0441
287.0551
kaempferol
m/z=213.0547
$-C_2H_2O_3$
$-C_8H_7O_4$
m/z=121.0285
m/z=287.0549
cyanidin
$-CH_2O_2$
$-C_8H_7O_3$
m/z=241.0496
m/z=137.0234

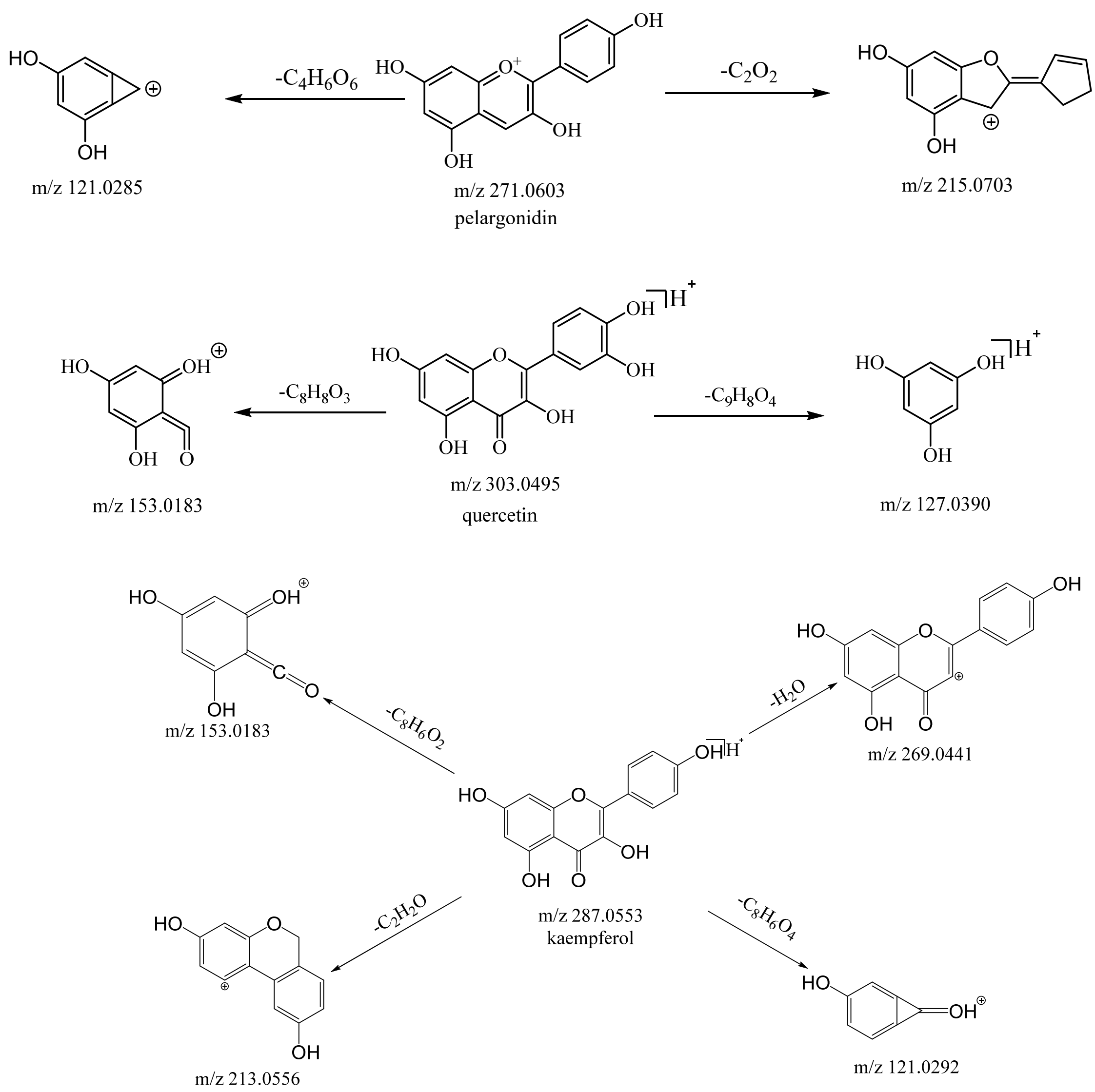

Figure S9. MS/MS spectra and fragmentation pattern of the main flavonoid aglycones in the hydrolysate.

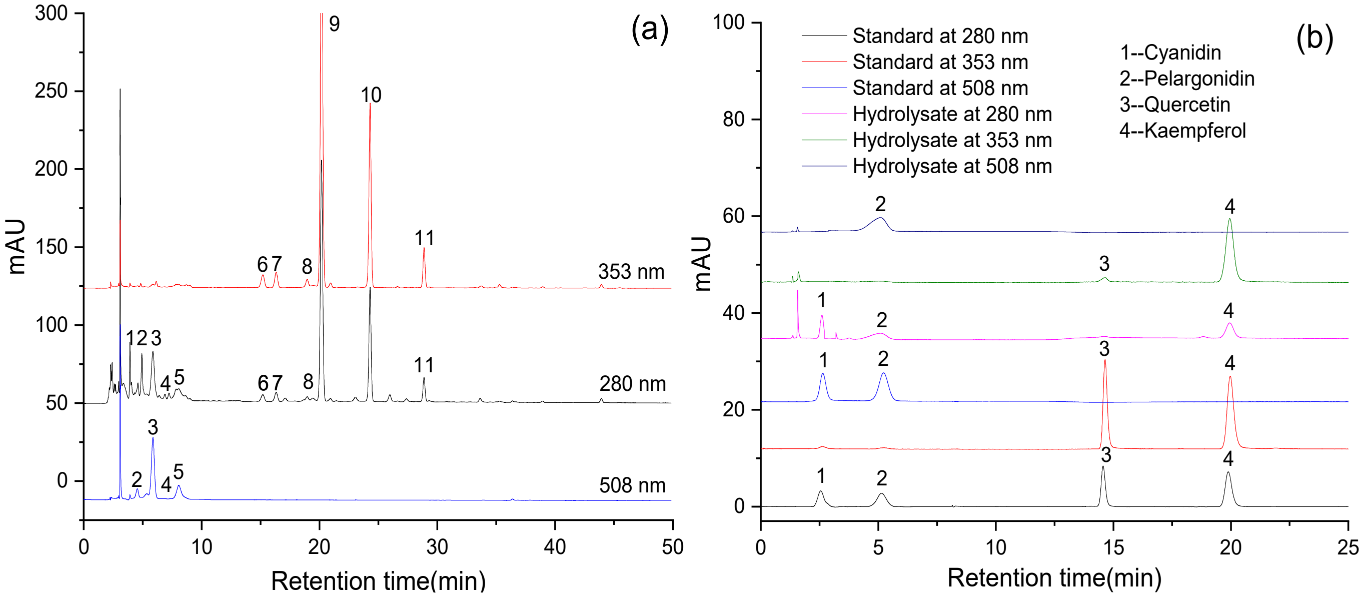

Figure S10. The chromatograms of the extract before (a) and after (b) UAAH hydrolysis by HPLC-DAD detection.

Table S1. The experimental results for Box-Behnken Design: salt concentration (X_1), extraction temperature (X_2), ultrasonic power (X_3) and solvent-to-solid ratio (X_4).

Run	Factors				Extraction yield
	X_1 (%, w/w)	X_2 (°C)	X_3 (%)	X_4 (mL/g)	(mg/g)
1	0 (18)	-1 (60)	0 (70)	1 (80)	27.42
2	0 (18)	0 (70)	0 (70)	0 (70)	37.26
3	1 (19)	0 (70)	1 (80)	0 (70)	33.48
4	0 (18)	0 (70)	-1 (60)	1 (80)	26.78
5	1 (19)	1 (80)	0 (70)	0 (70)	33.04
6	0 (18)	0 (70)	0 (70)	0 (70)	36.21
7	0 (18)	0 (70)	1 (80)	-1 (60)	28.74
8	0 (18)	1 (80)	-1 (60)	0 (70)	29.08
9	0 (18)	0 (70)	1 (80)	1 (80)	34.45
10	0 (18)	1 (80)	1 (80)	0 (70)	33.18
11	0 (18)	0 (70)	0 (70)	0 (70)	36.88
12	1 (19)	0 (70)	0 (70)	1 (80)	32.51
13	-1 (17)	0 (70)	1 (80)	0 (70)	29.49
14	0 (18)	0 (70)	-1 (60)	-1 (60)	28.47
15	1 (19)	-1 (60)	0 (70)	0 (70)	27.73
16	0 (18)	1 (80)	0 (70)	-1 (60)	30.25
17	0 (18)	0 (70)	0 (70)	0 (70)	36.19
18	1 (19)	0 (70)	0 (70)	-1 (60)	29.76
19	0 (18)	-1 (60)	0 (70)	-1 (60)	26.95
20	-1 (17)	0 (70)	0 (70)	1 (80)	30.18
21	-1 (17)	-1 (60)	0 (70)	0 (70)	26.36
22	0 (18)	-1 (60)	1 (80)	0 (70)	27.86
23	0 (18)	1 (80)	0 (70)	1 (80)	33.35
24	0 (18)	0 (70)	-1 (60)	0 (70)	25.21
25	-1 (17)	-1 (60)	0 (70)	-1 (60)	28.96
26	1 (19)	0 (70)	-1 (60)	0 (70)	26.58
27	-1 (17)	1 (80)	0 (70)	0 (70)	31.24
28	-1 (17)	0(70)	-1 (60)	0 (70)	27.54
29	0 (18)	0(70)	0 (70)	0 (70)	36.79
